# Regulatory effects of mangiferin on LPS‐induced inflammatory responses and intestinal flora imbalance during sepsis

**DOI:** 10.1002/fsn3.3907

**Published:** 2023-12-27

**Authors:** Bo‐tao Chang, Yang Wang, Wen‐lian Tu, Zhi‐qing Zhang, Yan‐fang Pu, Li Xie, Fang Yuan, Ying Gao, Ning Xu, Qi Yao

**Affiliations:** ^1^ Department of Postgraduate Guizhou University of Traditional Chinese Medicine Guiyang China; ^2^ Department of General Surgery The First People's Hospital of Yunnan Province, The Affiliated Hospital of Kunming University of Science and Technology Kunming China; ^3^ Department of Pharmacy The First People's Hospital of Yunnan Province, The Affiliated Hospital of Kunming University of Science and Technology Kunming China; ^4^ The First Affiliated Hospital, Guizhou University of Traditional Chinese Medicine Guiyang China; ^5^ Department of Clinical Laboratory The First People's Hospital of Yunnan Province, The Affiliated Hospital of Kunming University of Science and Technology Kunming China

**Keywords:** inflammation, intestinal flora, lipopolysaccharide, mangiferin, TLR4/myD88/NF‐κB

## Abstract

Studies suggest that mangiferin (MAF) has good therapeutic effects on chronic bronchitis and hepatitis. Also, it is one of the antiviral ingredients in *Anemarrhena asphodeloides* Bunge. However, its effect on the LPS‐induced inflammation and intestinal flora during sepsis remains unclear yet. In the present study, LPS‐stimulated inflammation RAW264.7 cells and LPS‐induced sepsis mice were used to evaluate the efficacy of MAF in vitro and in vivo. 16S rDNA sequencing was performed to analyze the characteristics of intestinal flora of the sepsis mice. It has been demonstrated that MAF (12.5 and 25 μg/mL) significantly inhibited protein expressions of TLR4, MyD88, NF‐κB, and TNF‐α in the LPS‐treated cells and reduced the supernatant TNF‐α and IL‐6 levels. In vivo, MAF (20 mg/kg) markedly protected the sepsis mice and reduced the serum TNF‐α and IL‐6 levels. Also, MAF significantly downregulated the protein expressions of TLR4, NF‐κB, and MyD88 in the livers. Importantly, MAF significantly attenuated the pathological injuries of the livers and small intestines. Further, MAF significantly increased proportion of *Bacteroidota* and decreased the proportions of *Firmicutes*, *Desulfobacterota*, *Actinobacteriota*, and *Proteobacteria* at phylum level, and it markedly reduced the proportions of *Escherichia‐Shigella*, *Pseudoalteromonas*, *Staphylococcus* at genus level. Moreover, MAF affects some metabolism‐related pathways such as citrate cycle (TCA cycle), lipoic acid metabolism, oxidative phosphorylation, bacterial chemotaxis, fatty acid biosynthesis, and peptidoglycan biosynthesis of the intestinal flora. Thus, it can be concluded that MAF as a treatment reduces the inflammatory responses in vitro and in vivo by inhibiting the TLR4/ MyD88/NF‐κB pathway, and corrects intestinal flora imbalance during sepsis to some degree.

## Graphical abstract

Key findings: MAF reduces LPS‐induced inflammatory reaction in vitro and in vivo; MAF exerts its anti‐inflammatory effect by inhibiting the TLR4/MyD88/NF‐κB pathway; MAF corrects intestinal flora imbalance during early stage of sepsis to some degree.

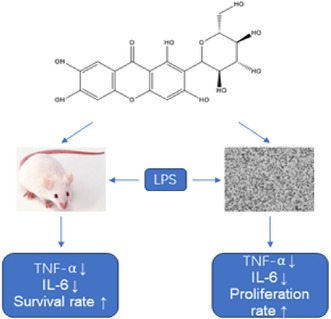

## 1 INTRODUCTION

Recently, sepsis is becoming a global public health problem. Also, it is a conspicuous cause of death due to infectious factors, with a mortality rate of up to 40% (Fleischmann et al., 2016). Commonly, sepsis is initiated by bacteria, fungi, viruses, and other pathogenic microbes. The pathogenesis is extremely complex, including the body's inflammatory reaction imbalance, coagulation, and immune disorders, mitochondrial function damage, and other pathophysiological processes (Musie et al., 2014), which eventually leads to systemic circulatory and organ dysfunction, and even develops to septic shock, threatening patient's life seriously (Labib, 2019).

As a pattern recognition receptor (PRR), TLR4 is recognized by LPS derived from gram‐negative (G^−^) bacteria. It activates MyD88 then NF‐κB and finally releases inflammatory cytokines including TNF‐α, interleukin‐1β (IL‐1β), and IL‐6 which play crucial roles in occurrence and development of sepsis (Roger et al., 2009).

During severe sepsis, the TLR4 signaling pathway mediates harmful and excessive inflammatory responses. Conversely, lack of TLR4 signaling weakens cytokine generation, improves hemodynamics, reduces tissue damage, and elevates survival rate. It has been confirmed that TLR4 deficiency improves immune paralysis by restoring a proinflammatory cytokine balance and attenuating Treg activity in a model of postseptic mice (Cao, Chai, et al., 2018). Compared with wild‐type mice, the TLR4 knockout decreased mortality rates, improved cardiac dysfunction, and reduced expressions of IL‐1β, IL‐6, and TNF‐α in heart tissues and decreased neutrophil infiltration in cecum ligation and puncture (CLP)‐induced sepsis mice (Zhou et al., 2018). Further, the deletion of TLR4 significantly attenuated LPS‐induced acute liver injury in mice (Chen, Tan, et al., 2021). Thus, it is a promising strategy to modulate TLR4 to treat sepsis (Kumar, 2020; Senousy et al., 2022).

Recently, it has been revealed that the mRNA and protein levels of MyD88, NF‐κB, and TNF‐α are significantly upregulated in a CLP‐induced septic peritonitis rat model at various time points after the surgery (Lee et al., 2022). Actually, the inhibition of the TLR4/NF‐κB signaling pathway attenuates sepsis and its related complications. For example, it has been confirmed that ulinastatin reduces LPS‐induced acute lung injury by suppressing activation of this pathway and reducing inflammatory mediators (Cao et al., 2018).

Commonly, intestine is one of the main target organs of sepsis. In this event, intestinal barrier dysfunction is a conspicuous characteristic feature (Hu et al., 2019; Yoseph et al., 2016) accompanied by changes in total amount and proportion of digestive tract flora including increases and decreases in species, excessive growth of a single strain, structural change in intestinal flora, decreases in specific anaerobic bacteria and increases in facultative anaerobic bacteria, decreases in beneficial symbiotic bacteria and increases in opportunistic pathogenic bacteria, and some pathogenic bacteria regarded as the main genera (Graspeuntner et al., 2019; Wan et al., 2018).

Currently, it has revealed a positive correlation between severity degree of sepsis and intestinal flora imbalance (Wang et al., 2022). Recently, a 4‐week probiotic treatment changes flora structure, increases fecal flora probiotics, and improves functional diversity of flora in patients with mild sepsis (Stadlbauer et al., 2019). In addition, fecal microbiota transplantation (FMT) has been applied to improve *Enterobacteriaceae* illness in experimental and clinical studies, whereas it increases death probability for resistant pathogenic bacteria (Kim et al., 2020; Wei et al., 2016).

Mangiferin (MAF) is a bisphenylpyrone flavonoid compound in some medicinal plants including *Mangifera indica* L., *Anemarrhena asphodeloides* Bge., *Belamcanda chinensis* (L.) DC., and others. Its chemical name is 2‐β‐D‐glucopyranosyl‐1,3,6,7‐tetrahydroxy‐9H‐xanthen‐9‐one and the molecular formula is C_19_H_18_O_11_ (Molecular weight: 422.340, CAS: 4773‐96‐0). Additionally, MAF is one of the effective ingredients of *Gentiana rhodantha Franch*. ex Hemsl.

Recently, a study shows that MAF significantly protects sepsis‐induced lung and kidney injuries by its anti‐inflammatory and antioxidant effects (Garrido et al., 2004; He et al., 2014; Zhang et al., 2020), which is associated with suppression of TNF‐α and nitric oxide (NO) production (Garrido et al., 2004), inhibition of NLRP3 inflammasome activation (Li, Xiong, et al., 2021), and upregulation of heme oxygenase‐1 (Gong et al., 2013). Up to now, no study has reported the regulatory effect of MAF on the TLR4/myD88/NF‐κB signaling pathway during sepsis, let alone its effect on the intestinal flora.

Based on the previous studies, this study investigated the interventive effect of MAF on LPS‐induced inflammatory responses in vitro and in vivo. Also, its regulatory effect on the intestinal flora imbalance at the early stage of sepsis was assayed. In view of this, it is expected to provide some evidences for the future application of this compound in the treatment of sepsis.

## 2 MATERIALS AND METHODS

### 2.1 Materials

MAF (HPLC: Purity ≥ 98%) was from Solarbio Life Sciences. The RAW264.7 cell line was purchased from Procell Life Science & Technology Co., Ltd. Dexamethasone (DEX) sodium phosphate injection was obtained from Huazhong Pharmaceutical Co., Ltd. The male SPF ICR mice were provided by Huabukang Biotechnology Co., Ltd. The product license number was SCXK 2019–0008, and the use license number was SYXK 2021–0005. The animal experiment was approved by the Ethnic Committee of Experiment Research Institute of Guizhou University of TCM. The TLR4 and myD88 primary antibodies were provided by Santa Cruz Biotechnology Inc. The NF‐κB and TNF‐α were from Proteintech Inc. The secondary anti‐mouse and anti‐rabbit antibodies were obtained from Beijing Zhongshan Golden Bridge. The mouse TNF‐α and IL‐6 ELISA kits were from Dakewe Biotech Co., Ltd.

### 2.2 Cell viability assay in status of MAF (Ren et al., 2018)

The RAW264.7 cells were cultured in a 96‐well plate (10^4^/well) in DMEM supplemented with 10% FBS and 1% penicillin–streptomycin. After cultured for 12 h, the medium was replaced by the serum‐free medium followed by adding 1‐μL drugs of various concentrations to coculture for another 24 h. After that, 10‐μL CCK‐8 was added to coculture for 2 h. Finally, the optical density (OD) value of the solution was detected at 450 nm by using a Mutiskan Go microplate reader (Thermo Scientific). The cell proliferation rate (%) = (OD_Treated_ – OD_Blank_) and (OD_Control_ – OD_Blank_) × 100%.

### 2.3 Western blotting analyses of TLR4, myD88, NF‐κB, and TNF‐α in LPS‐treated RAW264.7 cells (Cao et al., 2019)

The cells (10^5^/well) were seeded in a six‐well plate, cultured overnight, and then treated with LPS to reach a final concentration of 200 ng/mL. Next, MAF dissolved in 1‰ DMSO (Solarbio Life Sciences) was added to achieve final concentrations of 6.25, 12.5, and 25 μg/mL, and then cocultured for 4, 8, and 12 h, respectively. Correspondingly, DEX (4 μg/mL) was selected as a positive control. At the specific time points, the cell supernatants and precipitations were collected. The cell supernatants were stored at −80°C for ELISA assay. The cell precipitations were lysed by boiling 6 × loading buffer containing 0.54% sodium dodecyl sulfate (SDS) (Beyotime Biotechnology), ultrasonicated for 30 s on ice by using a LANYI‐650Y ultrasonic cell disrupter system (Lanyi Instrument Co., Ltd.) (22 ± 1 KHz, 5–150 w), and centrifuged at 13,000 *g* for 5 min to harvest the supernatants. The total proteins were then separated by 10% SDS–polyacrylamide gel electrophoresis (SDS‐PAGE) followed by semidry transfers onto 0.22‐μm PVDF membranes. Then, the membranes were blocked with 5% fat‐free milk at room temperature for 1 h. The primary anti‐TLR4 (1:1000), myD88 (1:2000), NF‐κB (1:2000), and TNF‐α (1:2000) antibodies were added to coculture for 2 h at room temperature. After that, the secondary anti‐mouse antibody (1: 6000) or anti‐rabbit antibody (1: 6000) was added to coculture at room temperature for another 1 h and then imaged with enhanced chemiluminescence (ECL) by using a ChemiDoc™ Imaging System (BIO‐RAD).

### 2.4 Supernatant ELISA assays of TNF‐α and IL‐6

The TNF‐α and IL‐6 levels in the collected supernatants were assayed by using the ELISA method provided by the instructions of the manufacturer.

### 2.5 Establishment of LPS‐induced sepsis mouse model (Wang et al., 2021) and treatments

A total of 60 male ICR mice weighing 20–22 g were randomly separated into six groups including a vehicle group, an LPS‐treated group, a dexamethasone (DEX) group (4 mg/kg, i.p), and three MAF groups (5, 10, and 20 mg/kg, i.g) (dissolved in 5% CMC‐Na) (*n* = 10). Except for the vehicle and the LPS groups, the animals in other groups received various treatments once a day and it lasted 5 days. LPS solutions (20 mg/kg) were intraperitoneally injected into the mice except for the vehicle ones 4 h after the last treatments. The mice in the vehicle group were injected with equal volumes of NS. The peripheral blood samples were collected by cutting trails to harvest the sera for the ELISA assay in six animals each group 6 h post the LPS injections. After the injections, animal survivals in the groups were recorded every day within 7 days. In addition, the diet, weight, and mental state of the animals were also observed. At the end of the experiment, the survival curves were plotted and the differences were compared among the groups. In addition, the livers and small intestines were immediately collected when the animals were dead. Parts of the livers were for western blotting assay. Other parts of the livers and small intestines were for pathological examination.

### 2.6 ELISA assays of serum TNF‐α and IL‐6

The TNF‐α and IL‐6 in the collected sera were assayed by using the ELISA method provided by the instructions of the manufacturer.

### 2.7 Western blotting analyses of TLR4, myD88, and NF‐κB proteins in livers (Tian et al., 2021)

About 100 mg of liver samples was ground for homogenates, then lysed by boiling 6 × loading buffer, ultrasonicated for 1 min on ice as described before, and centrifuged at 13,000 *g* for 5 min to harvest the supernatants. Next, the total tissue proteins were then separated by 10% SDS‐PAGE and then semidry transferred onto the PVDF membranes. The membranes were then blocked with 5% fat‐free milk for 1 h and cocultured with anti‐TLR4 (1:1000), myD88 (1:2000), and NF‐κB (1:2000) antibodies for 2 h at room temperature. After washing with TBST three times, the membranes were cocultured with anti‐mouse antibody (1:6000) or anti‐rabbit antibody (1:6000) for another 1 h. Finally, the membranes were imaged with ECL.

### 2.8 Immunohistochemistry assays of TLR4 and NF‐κB in livers (Clark & Torbenson, 2017)

Routinely, 4% paraformaldehyde was used to fix the liver tissues for 24 h. After that, the tissues were dehydrated and embedded in paraffin. The samples were then cut into 2‐μm‐thick sections consecutively followed by dewaxing by xylene I, II, and III for 10 min, respectively, hydration by absolute ethanol for 15 min, 95% ethanol for 10 min, and 80% ethanol for 10 min. Next, antigen repair was performed in sodium citrate solution at 90°C for 5 min in a microwave oven.

The sections were placed in 0.3% PBST solution for 20 min to disrupt the cellular membranes and washed with PBS three times. An immunohistochemistry assay kit (Zhongshan Golden Bridge Biotechnology Co. Ltd.) was used in this experiment. Appropriate amount of endogenous peroxidase was added to the sections in a wet box, incubated at room temperature for 10 min, rinsed three times with PBS, and then blocked with 5% sheep serum for 30 min at room temperature.

After that, the first antibodies TLR4 (1:100) and NF‐κB (1:100) were added, coincubated for 1 h at 37°C and washed with PBS three times and each 3 min. Subsequently, the secondary antibody was added and coincubated for 20 min at room temperature, then rinsed with PBS three times. Finally, 3, 3′‐diaminobenzidine (DAB) was added and incubated for about 3 min to terminate the reaction. Hematoxylin staining for 30–60 s was used for re‐staining. The positive immunostaining granules labeled with brownish yellow were observed under an inverted microscope. TLR4 is mainly located on the cellular membrane, and NF‐κB is primarily located in the cytoplasm. The positive cell number per unit area and the total cell number per unit area were, respectively, counted by calculating random five fields from each section. Positive expression rate was expressed as a ratio of positive cell number and total cell number.

### 2.9 Hematoxylin and eosin (H&E) staining of livers and small intestines (Kuang et al., 2021)

The collected liver and small intestine samples were fixed, dehydrated, and embedded.

Next, the tissues were dewaxed, and hydrated as well as the procedures for the immunohistochemistry assay. Next, the paraffin samples were consecutively cut into 2‐μm‐thick sections followed by H&E staining. Finally, the pathological changes especially in tissular edema, inflammation, and hemorrhage were observed using a DM750 microscope and imaging system (Leica).

### 2.10 Feces collection and 16S rDNA sequencing of intestinal flora

Male SPF ICR mice weighing 20–22 g were randomly allocated into five groups including a blank group, an LPS group, a DEX group (8 mg/kg, i.p), and two MAF groups (20 and 40 mg/kg) (dissolved in 5% CMC‐Na, i.g) (*n* = 8–10). The mice in the groups received various treatments for continuously 5 days and once a day. Four hours after the last treatments, 0.1 mL of LPS solutions was injected intraperitoneally into the mice (20 mg/kg) except for the vehicle controls.

About 6 h after the LPS injections, the feces were collected and transferred in 1.5‐ml sterile tubes. The feces samples were then used for 16S rDNA sequencing to analyze changes in the characteristics of the intestinal flora of the mice. The sequencing was performed in accordance with a previous study (Haas et al., 2011). Briefly, cetyltrimethylammonium bromide (CTAB) was used to extract total genome DNA from the feces samples. The concentration and purity of the extracted DNA were measured by 1% agarose gel electrophoresis. After that, PCR was performed to amplify 16S rRNA genes of distinct regions of 16S V3‐V4. Next, a Qiagen Gel Extraction Kit (Qiagen) was used to purify the mixture. Sequencing libraries were generated by using a TruSeq® DNA PCR‐Free Sample Preparation Kit (Illumina). After assessing the library quality, the library was sequenced to generate 250‐bp paired‐end reads by an Illumina NovaSeq platform (Bokulich et al., 2013).

The raw data were screened and some specific sequences were removed. The exclusion criteria included a fragment length shorter than 200 bp, a quality score less than 20, ambiguous bases or mismatch primer sequences, and barcode tags.

Operation taxonomy units (OTUs) were obtained by clustering rDNA sequence at 97% similarity and removing chimeric sequences using the UPARSE 7.1 software available at http://drive5.com/uparse/ (Edgar, 2013). Software tools including Cutadapt (v1.9.1), Qiime (1.9.1), Vsearch (1.9.6), and Cytoscape (3.9.0) were used for statistical analysis. The confidence threshold was defined at 80%. The similarity between different samples was assayed by the clustering analyses and principal component analysis (PCA) based on the OTU information from each sample.

### 2.11 Data presentation and statistics

The data were expressed as mean ± standard deviation. One‐way analysis of variance (ANOVA) was used to compare significance among the groups. *p* < .05 is considered significant.

## 3 RESULTS

### 3.1 Cell viability in absence or presence of LPS

**FIGURE 1:**
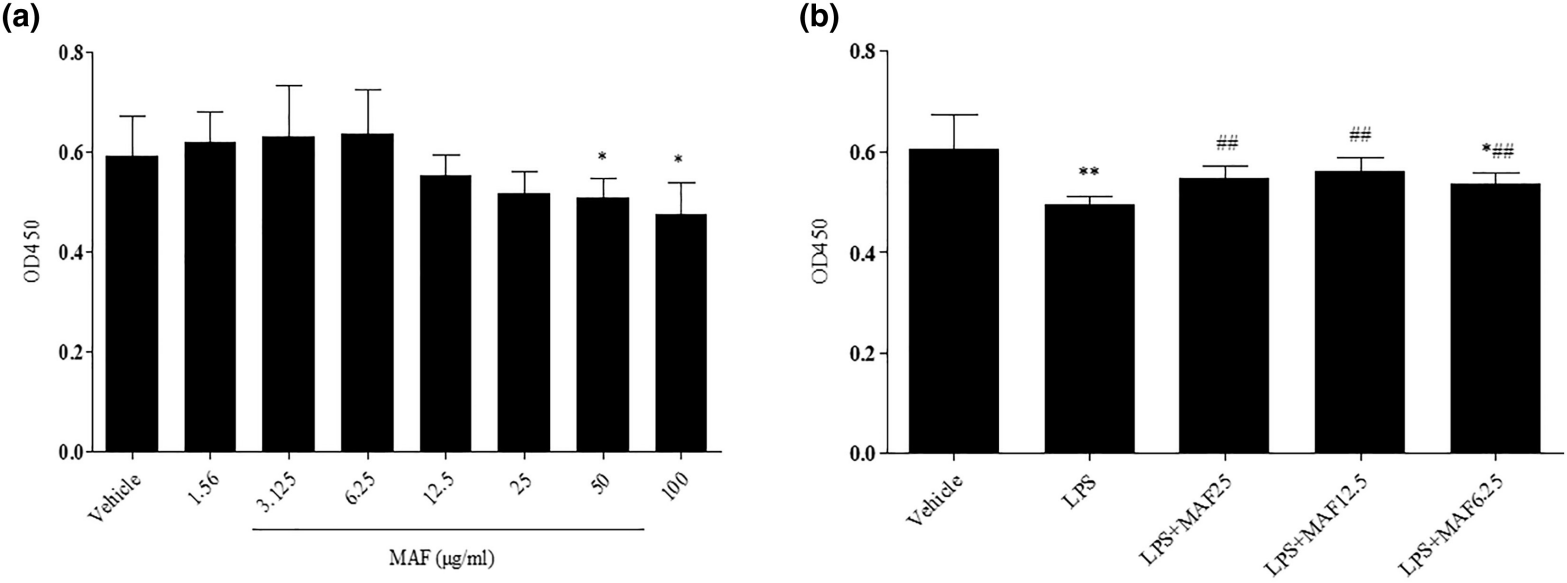
Effect of MAF on cell viability of RAW264.7 cells. (a) Cell viability in the absence of LPS (x̅ ± s, *n* = 3). *p < .05, **p < .01 vs. Vehicle. The cells were, respectively, cocultured with various concentrations of MAF (1.56–100 μg/mL) for 24 h followed by adding 10‐μL CCK‐8 to culture for another 2 h. The OD value of the solution was measured at 450 nm; (b) Cell viability in the presence of LPS (x̅ ± s, *n* = 3). *p < .05, **p < .01 vs. Vehicle; ^##^ *p* < .01 vs. LPS. The cells were treated with LPS (200 ng/mL) and MAF (6.25–25 μg/mL) for 24 h. Then, 10‐μL CCK‐8 was added to culture for another 2 h. After that, the OD value of the solution was measured at 450 nm.

It has been demonstrated that MAF less than 50 μg/mL had no significant toxicity for cellular growth compared to the vehicle. However, MAF at 50 and 100 μg/mL remarkably inhibited cellular proliferation with inhibition rates of 10.85% and 19.56%, respectively (Figure 1a). The LPS treatment (200 ng/mL) significantly slowed the cellular growth, while MAF (6.25–25 μg/mL) significantly retrieved the cellular growth with proliferation rates of 88.71%, 92.74%, and 90.84%, respectively (Figure 1b).

### 3.2 MAF inhibits TLR4/myD88/NF‐κB pathway in LPS‐treated cells

**FIGURE 2:**
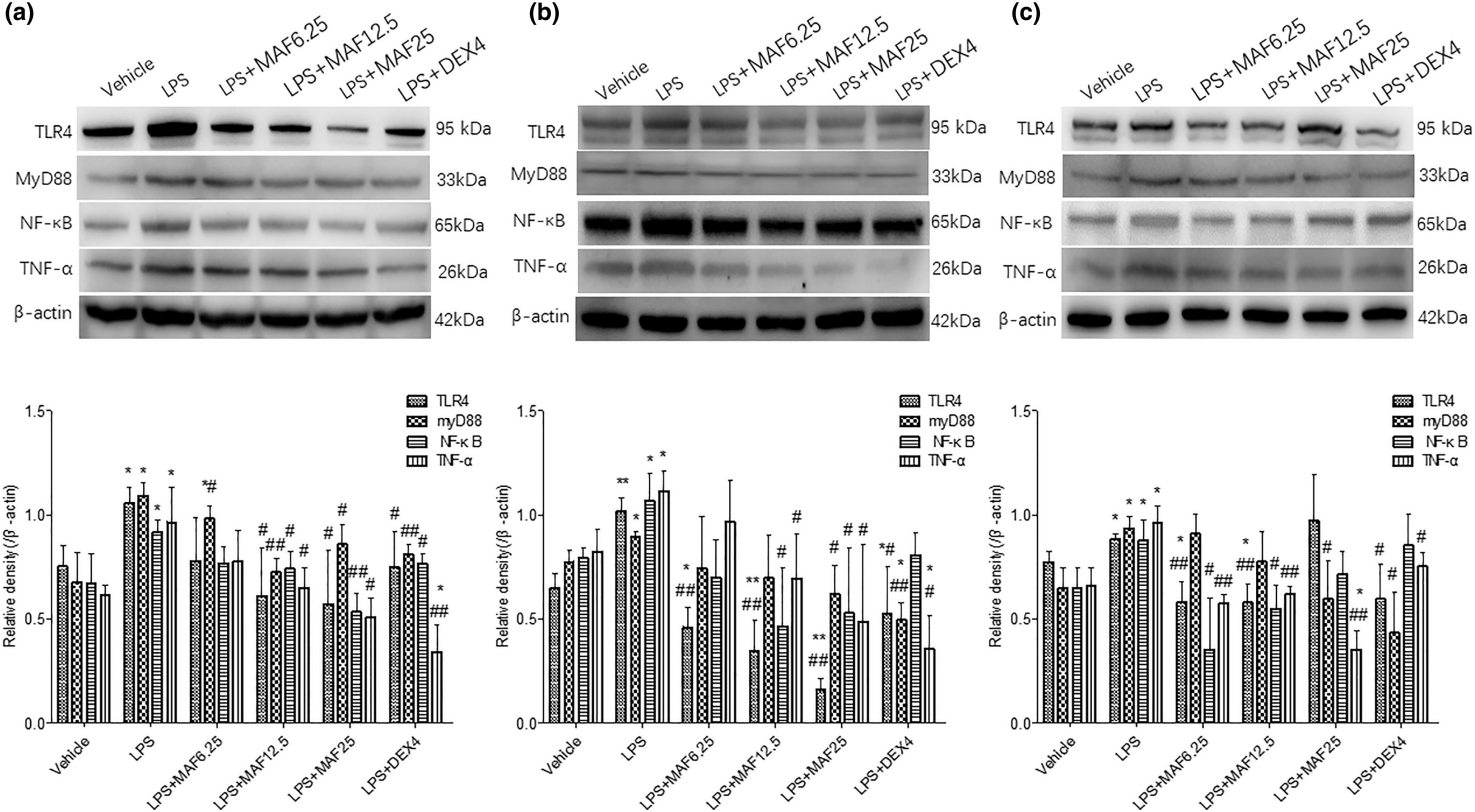
MAF downregulates the protein expressions of TLR4, myD88, NF‐κB, and TNF‐α in LPS‐stimulated cells at regular time points (x̅ ± s, *n* = 3). *p < .05, **p < .01 vs. Vehicle; ^#^ *p* < .05, ^##^ *p* < .01 vs. LPS. The cells were treated with MAF in the presence of LPS for 4 h (a), 8 h (b), and 12 h (c), respectively. The cell precipitations were collected and then lysed. The total proteins were then separated by using 10% SDS‐PAGE. After semidry transfer and coculture with the primary and secondary antibodies, the membranes were imaged with ECL.

The protein expressions of TLR4, myD88, NF‐κB, and TNF‐α in the LPS‐treated cells were significantly upregulated compared to the vehicle 4, 8, and 12 h after the LPS treatment (*p* < .05). Compared with the LPS treatment alone, MAF (6.25–25 μg/mL) remarkably downregulated the increased protein expressions of TLR4, myD88, NF‐κB, and TNF‐α 4, 8, and 12 h after the LPS treatment (*p* < .05) (Figure 2).

### 3.3 MAF reduces TNF‐α and IL‐6 levels in vitro and in vivo

Compared with the vehicle, LPS significantly upregulated supernatant TNF‐α and IL‐6 levels at 4, 8, and 12 h, respectively (*p* < .01). MAF at 6.25–12.5 μg/mL remarkably reduced the increased supernatant TNF‐α and IL‐6 levels compared to the LPS treatment alone at the regular time points (*p* < .05) (Tables 1 and 2).

**TABLE 1:** Supernatant TNF‐α level (pg/mL) at various time points (x̅ ± s, *n* = 3).

Treatment	Time (hours)		
	4	8	12
Vehicle	22.2 ± 4.3	59.4 ± 4.1	75.8 ± 7.4
LPS	301.0 ± 42.1**	467.0 ± 40.8**	1046.0 ± 83.9**
LPS + MAF6.25	176.6 ± 25.5**^#^	376.2 ± 64.2.1**	983.1 ± 146.0**
LPS + MAF12.5	132.6 ± 12.6**^##^	299.9 ± 18.9**^##^	795.5 ± 40.2**^##^
LPS + MAF25	107.8 ± 20.1**^##^	211.8 ± 42.5**^##^	507.4 ± 132.0**^##^
LPS + DEX4	96.5 ± 10.5**^##^	210.5 ± 45.7**^##^	470.0 ± 117.9**^##^

*Note*: **p < .01 vs. Vehicle; ^#^ *p* < .05, ^##^ *p* < .01 vs. LPS.

**TABLE 2:** Supernatant IL‐6 level (pg/mL) at various time points (x̅ ± s, *n* = 3).

Treatment	Time (hours)		
	4	8	12
Vehicle	29.2 ± 2.5	63.3 ± 7.4	70.9 ± 10.0
LPS	386.9 ± 63.1**	752.3 ± 118.9**	1280.6 ± 83.8**
LPS + MAF6.25	308.1 ± 50.4**	715.6 ± 119.4**	1150.8 ± 517.2**
LPS + MAF12.5	255.8 ± 43.3**^#^	513.7 ± 117.2**	824.7 ± 111.8**^##^
LPS + MAF25	181.7 ± 29.7**^##^	423.6 ± 25.8**^##^	689.1 ± 70.8**^##^
LPS + DEX4	179.6 ± 38.1**^##^	363.0 ± 107.3**^##^	528.7 ± 53.2**^##^

*Note*: **p < .01 vs. Vehicle; ^#^ *p* < .05, ^##^ *p* < .01 vs. LPS.

**FIGURE 3:**
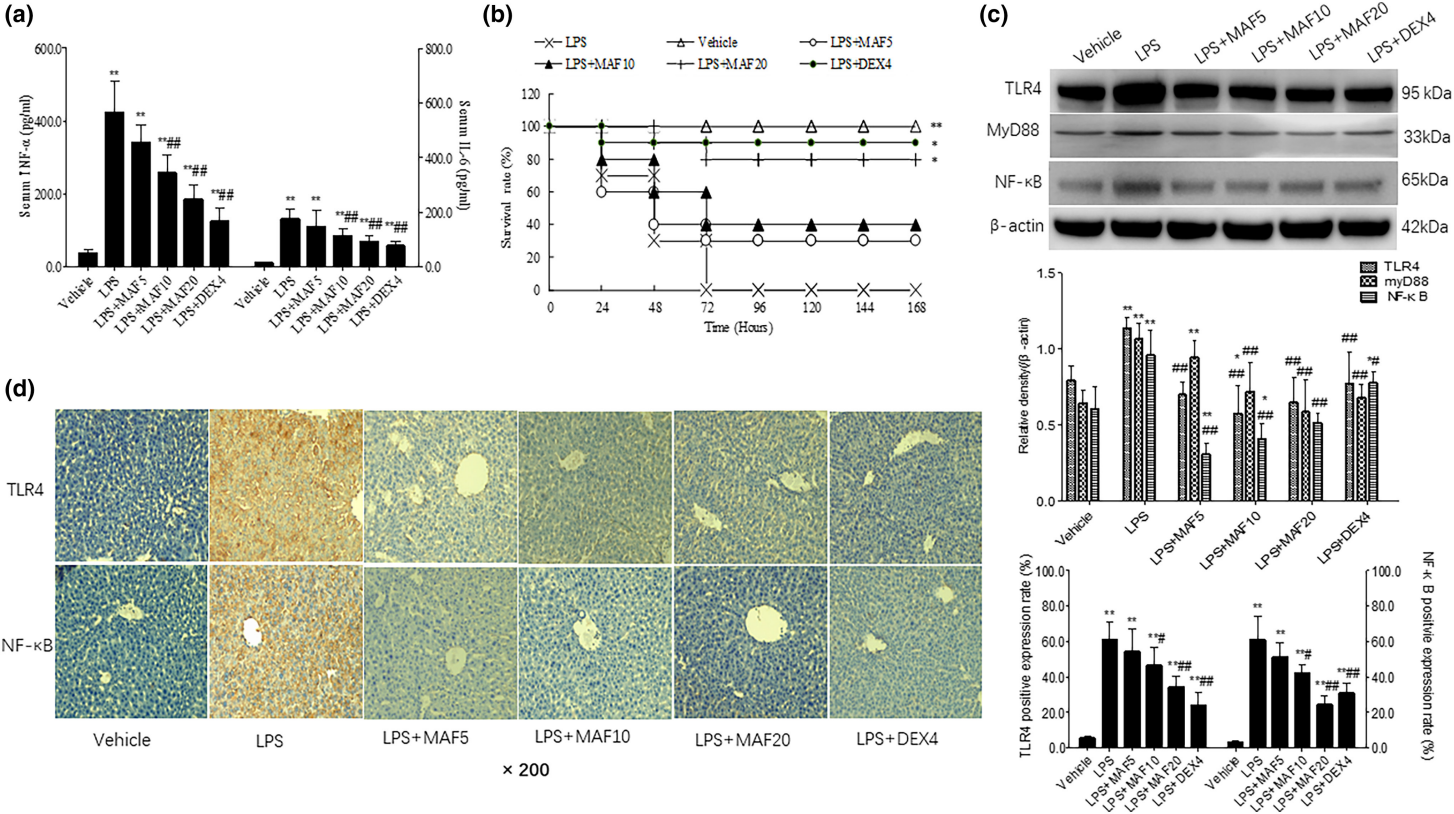
MAF protects LPS‐induced sepsis mice. (a) MAF decreases serum TNF‐α and IL‐6 levels in sepsis mice (x̅ ± s, *n* = 6). **p < .01 vs. Vehicle, ^#^ *p* < .05, ^##^ *p* < .01 vs. LPS. Before the LPS injections, the mice were, respectively, treated with MAF (5, 10, and 20 mg/kg) and DEX (4 mg/kg) for 5 days continuously and once a day. LPS (20 mg/kg) was intraperitoneally injected into the mice 4 h post the last treatments except for the vehicle controls. Then, the peripheral blood samples were collected by cutting tails. The serum TNF‐α and IL‐6 levels were assayed by using the ELISA method; (b) MAF increases the survival rate of the sepsis mice. *p < .05, **p < .01 vs. LPS. After the LPS injections, deaths of the animals in groups were recorded and plotted to draw a survival curve. The differences among groups were examined by Kaplan–Meier method; (c) MAF downregulates protein expressions of TLR4, MyD88, and NF‐κB in livers of sepsis mice (x̅ ± s, *n* = 6). **p < .01 vs. Vehicle, ^#^ *p* < .05, ^##^ *p* < .01 vs. LPS. The livers were immediately collected after the animals were dead. Parts of the livers were for western blotting analysis and others were for pathological analysis. The liver tissues were homogenized and then lysed. The total proteins were separated and transferred onto PVDF membranes. The membranes were blocked, and then cocultured with anti‐TLR4, myD88, and NF‐κB antibodies, respectively. After coculturing with the corresponding secondary antibodies, the membranes were imaged with ECL. (d) Immunohistochemical analysis of TLR4 and NF‐κB in livers (x̅ ± s, *n* = 6). **p < .01 vs. Vehicle, ^#^ *p* < .05, ^##^ *p* < .01 vs. LPS. The liver samples were fixed, dehydrated, embedded, and then cut into sections followed by dewaxing, hydration, and antigen repair. Anti‐TLR4 and NF‐κB antibodies were added and then cocultured with the secondary antibodies. After that, DAB was used to terminate the reaction. The positive granules labeled with brownish yellow were observed under an inverted microscope.

Similarly, the serum TNF‐α and IL‐6 levels were significantly higher in the model group than those in the vehicle group, while the MAF pretreatments (10 and 20 mg/kg) markedly reduced the increased serum TNF‐α and IL‐6 levels in the sepsis mice, and the differences were significant (*p* < .05) (Figure 3a).

### 3.4 MAF protects mice from lethal LPS challenge

Increased respiratory rate, decreased diet, lusterless hair, and wet watery feces occurred in the LPS‐induced sepsis mice. The animals began to die after the LPS injections. Death of the major animals was from 24 to 48 h after the injections, and all the animals in the model group died within 72 h (Figure 3b). It showed that the MAF pretreatment (20 mg/kg) significantly elevated the survival rate of the sepsis mice compared to the model control (*p* < .05) (Figure 3b).

### 3.5 MAF downregulates protein expressions of TLR4, myD88, and NF‐κB in livers

As the liver is one of the key target organs during sepsis, the proteins of TLR4, myD88, and NF‐κB in the livers were assayed to clarify the role of this pathway in the LPS‐induced sepsis. It showed that the protein expressions of TLR4, myD88, and NF‐κB were, respectively, higher in the model group than those in the vehicle group (*p* < .05). The MAF pretreatments (10 and 20 mg/kg) remarkably downregulated the increased expressions of these proteins in the livers compared to the model (Figure 3c).

### 3.6 MAF reduces positive expressions of TLR4 and NF‐κB in livers

Also, the immunohistochemistry assay showed that the positive expressions of TLR4 and NF‐κB proteins were, respectively, higher in the LPS‐treated group than those in the vehicle group (*p* < .01). Compared with the model, MAF (10 and 20 mg/kg) remarkably reduced the positive expressions of TLR4 and NF‐κB proteins in the livers (Figure 3d), which was consistent with the western blotting results in vivo.

### 3.7 MAF attenuates pathological injuries of livers and small intestines

**FIGURE 4:**
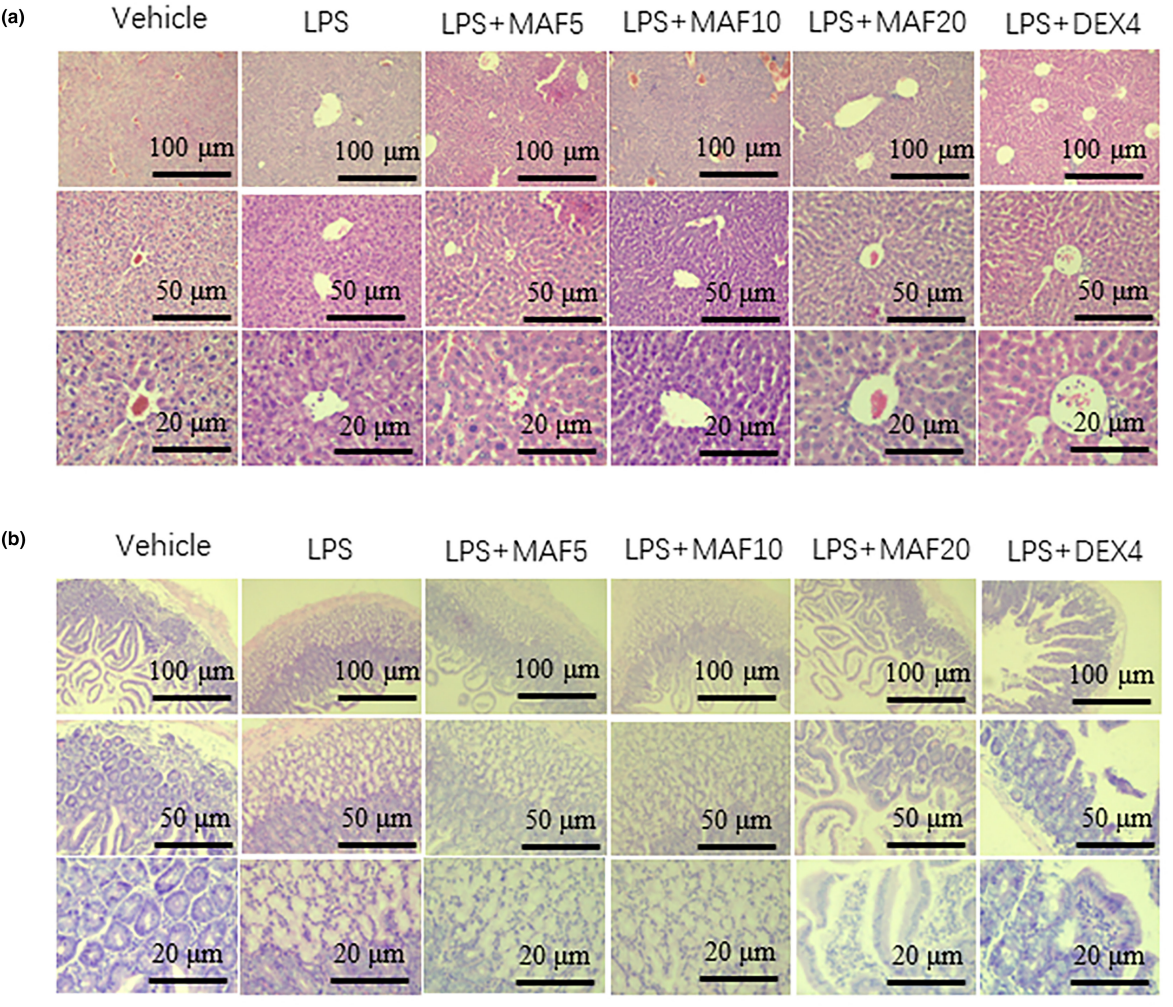
Pathological injuries of livers and small intestines of sepsis mice. The collected livers were fixed, dehydrated, and embedded. After that, the tissue samples were cut into sections. Next, the sections were dewaxed and hydrated followed by staining with H&E. The pathological changes especially edema, inflammation, and hemorrhage in the tissues were observed under an inverted microscope.

Clear and complete structures of hepatic lobules, cord‐like arrangements of hepatocytes, clear nuclear structures, and no necrosis or no inflammatory lesions were found in the livers of the vehicle group. However, the LPS treatment resulted in disordered arrangements, destroyed structures, and obvious swelling and bubble‐like changes of the hepatocytes, accompanied by necrosis and death of the partial cells, and inflammatory infiltrations (Figure 4a). MAF (5–20 mg/kg) in varying degrees improved damaged hepatic structure, alleviated inflammatory infiltration and liver congestion, and reduced cellular vacuolar degeneration and necrosis (Figure 4a).

The LPS treatment caused intestinal edema, inflammatory cell infiltration, and loss of intestinal epithelial integrity. Further, the arrangement of the intestinal glands in the cecal tissue was irregular, a large number of epithelial cells were swollen, and the cytoplasm was loose and lightly stained (Figure 4b). Actually, high‐dose MAF (20 mg/kg) significantly reduced intestinal edema and inflammatory cell infiltration, and restored intestinal epithelial integrity of the small intestines (Figure 4b).

### 3.8 MAF does not improve α diversity of intestinal flora

**FIGURE 5:**
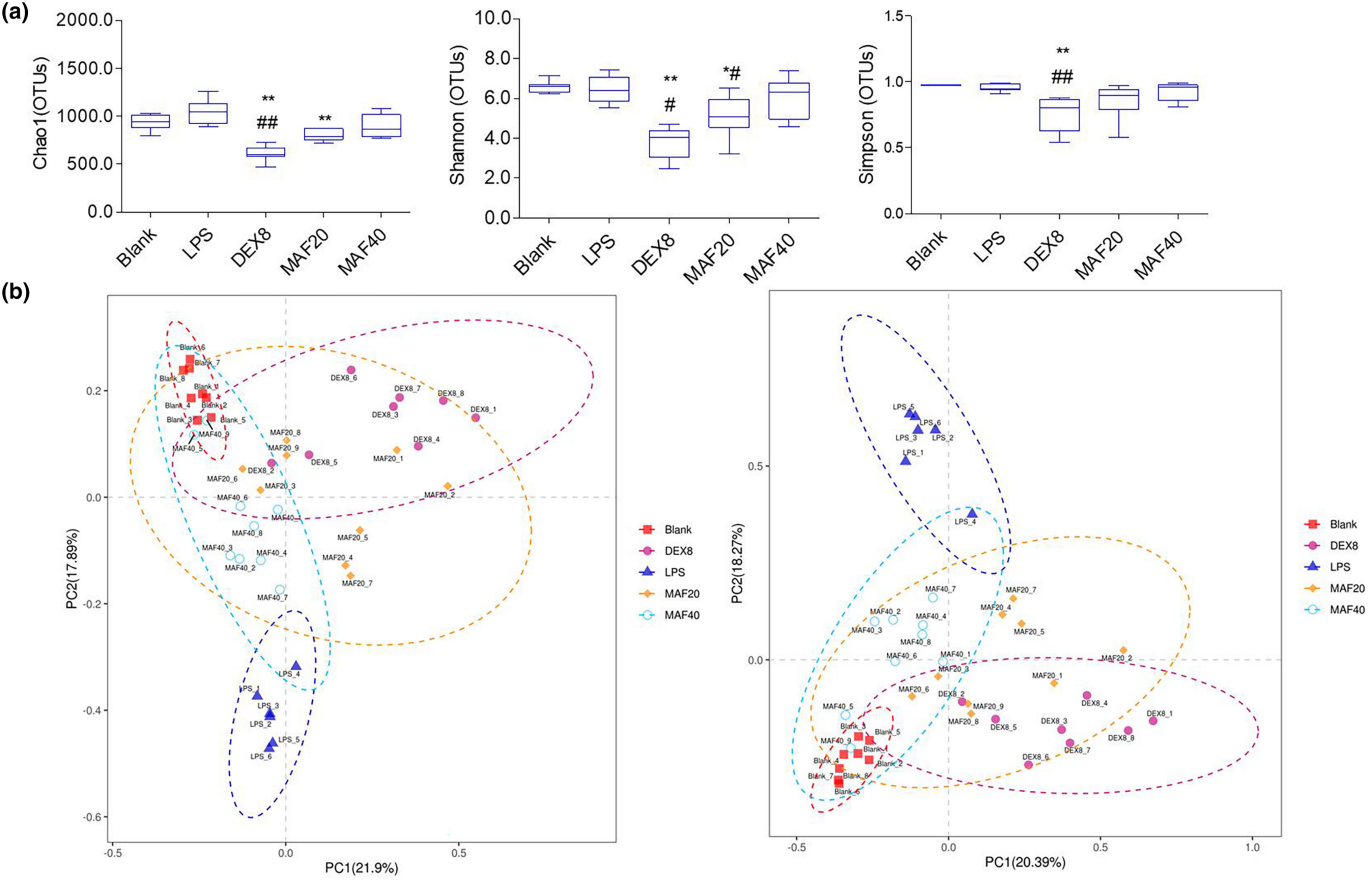
α and β diversity of intestinal flora in sepsis mice (x̅ ± s, *n* = 6–9). (a) The feces samples of the sepsis mice were collected, and 16S rDNA sequencing was then conducted. α diversity indices including Chao1, Shannon, and Simpson were assayed among groups to compare the differences among the groups; (b) PCoA assay was used to assay the similarity among the clusters.

There were no significant differences in Chao1, Shannon, and Simpson between the blank group and the LPS‐treated group (*p* > 0.05). Compared with the LPS treatment, DEX (8 mg/kg) and low‐dose MAF remarkably reduced these three indices (*p* < .05). In addition, no significant differences were found in these indices between the LPS‐treated group and the high‐dose MAF group (*p* > 0.05) (Figure 5a).

Principal coordinate analysis (PCoA) and similarity analysis (Anosim) were used to assay the similarity among the clusters by R language programming. It showed good sample separation after the principal coordinate analysis and similarity analysis, and β diversity by PCoA showed a clear clustering between the LPS group and the blank group, the MAF group, DEX group (Figure 5b).

### 3.9 MAF improves intestinal flora composition at phylum and genus levels, and affects metabolism‐related pathways

**FIGURE 6:**
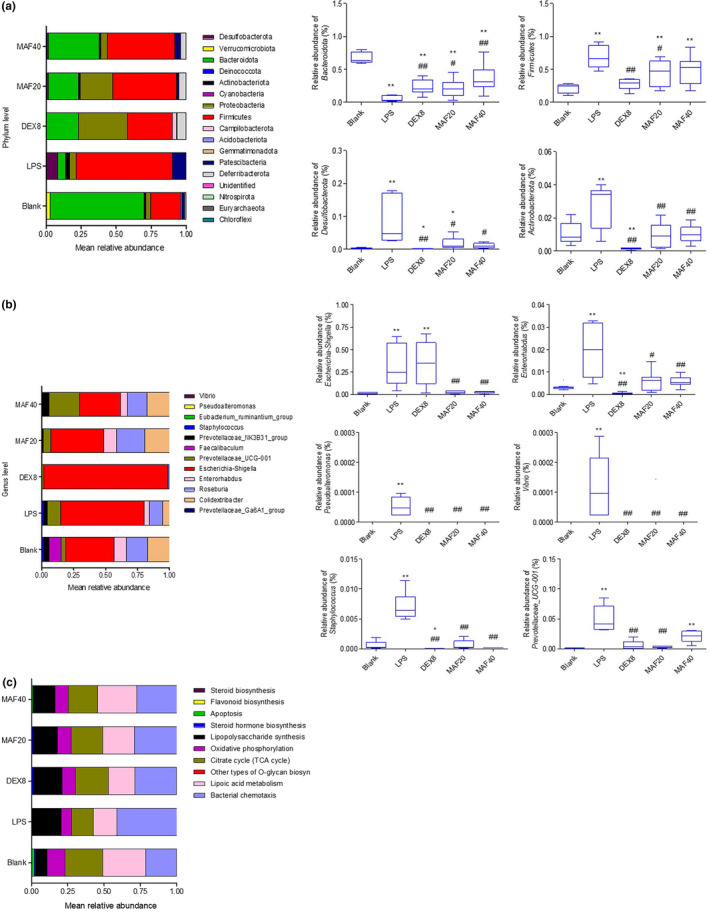
Intestinal flora composition and metabolism pathways. (a) Intestinal flora composition at phylum level (x̅ ± s, *n* = 6–9). *p < .05, **p < .01 vs. Blank; ^#^ *p* < .05, ^##^ *p* < .01 vs. LPS. The differences in the proportions of *Bacteroidota*, *Firmicutes*, *Desulfobacterota*, and *Actinobacteriota* were compared at phylum level among groups. (b) Intestinal flora composition at genus level (x̅ ± s, *n* = 6–9). *p < .05, **p < .01 vs. Blank; ^#^ *p* < .05, ^##^ *p* < .01 vs. LPS. The differences in the proportions of *Escherichia‐Shigella*, *Enterorhabdus*, *Pseudoalteromonas*, *Vibrio*, *Staphylococcus*, and *Prevotellaceae*_UCG‐001 were compared at genus level among groups. (c) Pathway analysis of intestinal flora among groups.

The intestinal flora composition in the blank group was mainly *Bacteroidota*, *Firmicutes*, *Proteobacteria*, *Desulfobacterota*, and *Patescibacteria* at phylum level. Compared with the blank control, the LPS treatment significantly decreased the proportion of *Bacteroidota* and increased the proportions of *Firmicutes, Desulfobacterota*, and *Actinobacteriota* (*p* < .05). DEX (8 mg/kg) remarkably increased the proportion of *Bacteroidota* and reduced the proportions of *Firmicutes*, *Desulfobacterota*, *Actinobacteriota*, and *Proteobacteria* compared to the LPS treatment (*p* < .05). The proportions of *Bacteroidota* in the MAF‐treated groups (20 and 40 mg/kg) were higher than that in the LPS‐treated group (*p* < .05). Meanwhile, the MAF pretreatments significantly downregulated the proportions of *Firmicutes*, *Desulfobacterota*, and *Actinobacteriota* compared to the LPS treatment (*p* < .05) (Figure 6a).

The LPS treatment significantly upregulated the proportions of *Escherichia‐Shigella*, *Enterorhabdus*, *Pseudoalteromonas*, *Vibrio*, *Staphylococcus*, and *Prevotellaceae*_UCG‐001 in the intestinal flora. Except for *Escherichia‐Shigella*, DEX (8 mg/kg) remarkably reduced the proportions of these five bacteria above compared to the LPS treatment (*p* < .01). It was found that the proportions of these six pathogenic bacteria were lower in the MAF‐treated groups (20 and 40 mg/kg) than those in the LPS‐treated group (*p* < .05), suggesting some antipathogenic bacterial activity of this compound (Figure 6b).

It showed that compared with the LPS treatment, MAF (20 and 40 mg/kg) significantly enhanced citrate cycle (TCA cycle), lipoic acid metabolism, oxidative phosphorylation, steroid hormone biosynthesis and apoptosis, and reduced bacterial chemotaxis, fatty acid biosynthesis, peptidoglycan biosynthesis (Figure 6c), which suggested the regulatory effect of MAF on these metabolism‐related pathways during sepsis.

## 4 DISCUSSION

Recently, studies have reported that MAF protects sepsis‐associated acute lung and kidney injuries via inhibiting inflammatory responses to oxidative stress (He et al., 2014; Zhang et al., 2020). The mechanisms included inhibition of NLRP3 inflammasome activation and upregulation of HO‐1 (Gong et al., 2013; Li, Xiong, et al., 2021). However, up to now, the anti‐inflammatory effect of this compound has not been systemically documented let alone its regulatory effect on the intestinal flora during sepsis.

Macrophages are the main participants in the body's inflammatory response. When the body undergoes an inflammatory reaction, macrophages will engulf pathogens and form antigen–antibody complexes by binding to the corresponding antibodies, ultimately eliminating the invading pathogens (Shapouri‐Moghaddam et al., 2018). Macrophages are polarized under various stimuli, which exacerbates inflammatory responses in the body (Juan et al., 2021). The LPS treatment induces macrophages to polarize toward M1 type and to exert immune effects (Chen, Liu, et al., 2021; Patoli et al., 2020), leading to the release of proinflammatory factors TNF‐α and IL‐6 (Oishi & Manabe, 2018). Also, LPS stimulates the product of NO, exacerbates cell damage, and results in various inflammatory diseases threatening the body's health seriously (Dickson & Lehmann, 2019; Page et al., 2022).

In the present study, MAF significantly inhibited LPS‐induced inflammatory responses in the RAW264.7 cells and CLP‐induced sepsis in the mice. In vitro, MAF significantly inhibited the increased protein expressions of the TLR4, MyD88, NF‐κB, and the downstream cytokines TNF‐α and IL‐6 in the LPS‐treated cells at the regular time points. In vivo, MAF markedly protected the mice from the LPS challenge, and reduced serum TNF‐α and IL‐6 levels and the protein expressions of TLR4, MyD88, and NF‐κB in the livers. These findings were similar to some previous studies (Li, Xiong, et al., 2021; Zhang et al., 2020). Both Zhang et al. (2020) and Li, Xiong, et al. (2021) found that MAF inhibited the activation of NF‐κB in the LPS‐induced sepsis. Thus, NF‐κB is considered to be a candidate target of MAF.

NF‐κB plays a role in inflammatory responses. Actually, the transcription and expression of proinflammatory cytokines are regulated by NF‐κB mainly activated by the transmembrane signaling transporter protein TLR4 (Chen et al., 2022). TLR4 can regulate NF‐κB dependent and independent of the MyD88 pathway. It recognizes external inflammatory stimuli and transfers external signals to the intracellular adapter protein MyD88 that recruits and phosphates interleukin‐1 receptor‐associated kinase 4 (IRAK4), which ultimately causes NF‐κB phosphorylation and its translocation into the nucleus, thereby promoting the occurrence and development of inflammatory mediators (Park et al., 2015).

Liver is one of the affected target organs during sepsis, and it is also closely related to the prognosis. Liver dysfunction after sepsis is an independent risk factor for MODS and sepsis‐associated death, which raises a huge challenge to clinical diagnosis and treatment (Woźnica et al., 2018). A study has revealed that inhibition of the TLR4/NF‐κB pathway decreases liver injury in CLP‐induced sepsis rats, which suggests a participation of this pathway in sepsis‐induced liver injury (Lee et al., 2022).

Our study showed that MAF significantly downregulated the expressions of TLR4, MyD88, and NF‐κB and reduced the levels of the proinflammatory cytokines in vitro and in vivo. Meanwhile, MAF protected the LPS‐induced sepsis mice and attenuated the liver and small intestinal injuries. Based on these findings above, it can be concluded that MAF exerts its anti‐inflammatory effect via inhibiting the TLR4/MyD88/NF‐κB pathway.

As a common target organ of sepsis, the epithelial mucosal damage of small intestine is an initiating factor for the occurrence and development of sepsis. During this period, inflammatory response of the intestinal mucosal epithelium is overactivated and edematous, resulting in disruptions of the intestinal epithelial cell membranes and intercellular connections, which leads to increased intestinal permeability and intestinal barrier functional damage (Chen et al., 2019). Simultaneously, the intestinal bacteria migrate into the circulatory system, releasing toxins causing SIRS and even MODS.

Previously, the Lancet journal reported that intestinal flora imbalance was closely related to the development of sepsis (Wetter et al., 1994). Intestinal flora imbalance refers to disturbance caused by changes in composition, distribution, and activity of the intestinal flora. The pathogenesis for the intestinal flora imbalance during sepsis mainly includes abnormal intestinal immune function, inflammatory and oxidative stress reactions in upper intestine, intestinal microcirculation disorders, and decreases in short‐chain fatty acids (SCFAs) (Yang et al., 2021).

Under normal conditions, 90% of intestinal bacteria are *Bacteroidetes* and *Firmicutes*, and others include *Archaea*, *Proteus bacillus vulgaris*, *Actinomycetes*, etc. When sepsis occurs, internal environment of the intestine is disrupted, and inherent gut flora also undergoes significant changes. The main manifestations include changes in gut flora types, flora structure, proportions of facultative anaerobic bacteria, specialized anaerobic bacteria, beneficial symbiotic bacteria, growth of a certain single strain, and opportunistic pathogens (Graspeuntner et al., 2019). Further, degree of the intestinal flora imbalance is correlated with the severity of sepsis positively (Adelman et al., 2020; Cabrera‐Perez et al., 2017). In addition, high‐level LPS is thought to be an independent risk factor for the intestinal flora imbalance at early stage of sepsis.

Our findings showed that MAF did not improve α diversity of the intestinal flora including the Chao1, Shannon, and Simpson indices between the blank group and the LPS‐treated group, which was some coherent with a study on intestinal flora at early stage of LPS‐induced sepsis (Li, Lin, et al., 2021), but it was some contradictory to changes in intestinal flora in a CLP‐induced sepsis mouse model from 6 h to 3 days (Zhao et al., 2021). We speculated that the differences might be due to the duration of the observation time and the modeling methods.

In addition, MAF significantly increased the proportion of *Bacteroidota* and decreased the proportions of *Firmicutes*, *Desulfobacterota*, and *Proteobacteria* at the phylum level. Meanwhile, MAF reduced the proportions of pathogenic bacteria, such as *Escherichia‐Shigella*, *Pseudoalteromonas*, *Staphylococcus*, etc., at the genus level. It suggested that MAF exerted its antisepsis effect in vivo partly via inhibiting the growth of some pathogenic bacteria and promoting the growth of some beneficial bacteria. Moreover, MAF enhanced the TCA cycle to inhibit adhesive invasive bacteria and reduced their virulence, increased lipoic acid metabolism to accelerate the clearance of the metabolites, elevated oxidative phosphorylation level to provide energy for the body, and reduced bacterial chemotaxis, decreased fatty acid and peptidoglycan biosynthesis to eliminate the toxins.

In summary, MAF ameliorates LPS‐induced inflammatory responses in vitro and in vivo by regulating the TLR4/MyD88/NF‐κB pathway. Moreover, MAF improves the intestinal flora imbalance in the LPS‐induced sepsis mice by inhibiting some pathogenic bacteria mainly via providing energy, reducing toxic biosynthesis, increasing metabolism, etc. In future study, experimental animals with TLR4 knockout are encouraged to explore the mechanism of MAF against sepsis more deeply.

All these findings provided some experimental evidence for the application of MAF in the treatment at early stage of sepsis in the future.

## AUTHOR CONTRIBUTIONS

**Bo‐tao Chang:** Investigation (equal). **Yang Wang:** Investigation (equal). **Wen‐lian Tu:** Formal analysis (equal). **Zhi‐qing Zhang:** Formal analysis (equal). **Yan‐fang Pu:** Data curation (equal). **Li Xie:** Data curation (equal). **Fang Yuan:** Data curation (equal). **Ying Gao:** Funding acquisition (equal); project administration (equal). **Ning Xu:** Project administration (equal). **Qi Yao:** Funding acquisition (equal); methodology (equal); writing – original draft (equal); writing – review and editing (equal).

## FUNDING INFORMATION

This work was supported by grants from National Natural Science Foundation of China (No. 81960795 and 81960746), Technology Cooperation Project of Guiyang Science & Technology Bureau and the First Affiliated Hospital of Guizhou University of TCM (2019‐9‐2‐4), 2022 High‐Level Talent Research Project of Yunnan Provincial Health Commission (2023‐KHRCBZ‐A02), and Open Project for Construction Unit of Clinical Pharmacy Center of Yunnan Province (2023YJZX‐YX11).

## CONFLICT OF INTEREST STATEMENT

All the authors declared no conflict of interest in this study.

## ETHICS STATEMENT

All the animal procedures complied with the Guide for the Care and Use of Laboratory Animals of the US National Institutes of Health and were approved by the Experimental Research Center of Guizhou University of TCM (GZUTCM‐LL‐ 20220095).

## Data Availability

The data in this study are deposited in the figshare repository, and the accession number is 10.6084 /m9. figshare.24588099.
